# Molybdenum as a biodegradable bone implant material assessed by ISO 10993-5/-12-compliant cytocompatibility testing and transcriptomic profiling in a human osteoblast cell line

**DOI:** 10.1038/s41598-026-56071-x

**Published:** 2026-07-02

**Authors:** Vanessa Curtaz, Hannah R. Richter, Maxine Kick, Peter Quadbeck, Fabian J. Eber

**Affiliations:** Hochschule Offenburg, Badstr. 24, 77652 Offenburg, Germany

**Keywords:** Biological techniques, Biotechnology, Cell biology, Materials science

## Abstract

Molybdenum is increasingly explored as a metallic implant material for bone regeneration owing to its high mechanical strength, controlled degradation behavior, and corrosion resistance. This study evaluated the cytocompatibility of metallic molybdenum using the osteoblast cell line hFOB 1.19 in both extract-based and direct-contact viability assays, conducted in strict accordance with ISO 10993-5/-12 to obtain valuable high-quality preclinical data. Molybdenum extracts demonstrated high cytocompatibility across all tested dilutions, with cell viability falling below the 70% ISO threshold only for the undiluted extract, indicating a mild concentration-dependent effect. In direct-contact assays, molybdenum preserved normal cell morphology and monolayer integrity comparable to titanium. In contrast, magnesium induced pronounced cytotoxic effects in both assay formats. All reference materials elicited the expected responses; however, gamma irradiation abolished the cytotoxicity of polyurethane containing zinc diethyldithiocarbamate, highlighting the critical importance of accounting for sterilization-induced material changes. Complementary transcriptomic analysis revealed upregulation of gene sets associated with immunostimulatory signaling, aerobic energy metabolism, and protein biosynthesis, while expression of osteogenic differentiation markers remained unimpaired. Together, these results demonstrate that cytotoxic effects of metallic molybdenum occur only at extract concentrations in the millimolar range, thereby establishing a sound preclinical basis for the further development of molybdenum-based biomaterials.

## Introduction

Bone defects arising from trauma, surgical resection, or infection can severely impair physiological function and tissue integrity, presenting major reconstructive challenges that demand both mechanical stability and biological integration. This is especially true in the craniomaxillofacial (CMF) region, where such defects additionally compromise vital functions such as mastication, swallowing, and respiration^1^. Autologous bone grafts remain the clinical gold standard owing to their excellent biological integration^2,3^, but their use is constrained by donor site morbidity, limited availability, and difficulties in shaping complex geometries^4^. Synthetic alternatives each address some but not all of these limitations: Poly-L-lactic acid (PLA) offers bioresorbability but generates acidic degradation products that trigger inflammation and fibrosis^5–7^. Magnesium (Mg) alloys are capable of complete biodegradation and, in the form of the alloy WE43 - containing approximately 4 wt% yttrium and about 3 wt% neodymium together with other heavy rare earth elements - is already used in approved medical devices such as stents and bone anchor screws. However, the corrosion behavior of magnesium can be difficult to control, often characterized by relatively rapid and sometimes localized degradation, hydrogen evolution, and transient increases in local alkalinity, which may affect mechanical stability and tissue response depending on the clinical context^8–11^. Titanium (Ti), although widely regarded for its superior mechanical strength and biocompatibility, is not biodegradable and therefore commonly necessitates secondary implant removal^12–14^. No current material successfully combines metallic strength with controlled resorption and favorable biological performance^15^.

Molybdenum (Mo) has recently emerged as a material capable of bridging the gap between rapidly degrading magnesium alloys and non-resorbable titanium implants. This offers uniform biodegradation alongside robust mechanical performance^16–18^. Upon degradation, Mo forms soluble molybdate ions (MoO₄²⁻) that are efficiently cleared renally^19^. Physiological serum molybdenum concentrations in adults are reported to be below 1 µg/L (< 10 nmol/L)^20^, and controlled human intake studies have demonstrated that even substantially elevated systemic exposure results in dose-dependent urinary excretion without clinical adverse effects, indicating effective physiological regulation^21^. Nevertheless, despite safe systemic clearance, local cytocompatibility at the bone-implant interface requires dedicated evaluation, as initial ion release concentrations may transiently exceed plasma levels and potentially impair osteoblast function. Previous in vitro work has provided encouraging initial evidence: LC_50_ values exceeded 7 mM in osteoblast-like MG63 cells^22^ and no adverse inflammatory response was observed at relevant exposure levels^17^. Both in vitro and in vivo studies further support the biocompatibility of metallic Mo, with positive tissue responses in rat models^17,23^ and promising results in CMF applications^18^. However, elevated molybdenum exposure has been shown to lead to renal toxicity with copper deficiency and oxidative stress discussed as underlying mechanisms of action^19^. Importantly, cytocompatibility of Mo appears concentration-dependent, with cytotoxic effects on osteoblasts reported only at supraphysiological molybdate concentrations above 0.1 mM^22^. Prior in vitro studies employed non-standardized exposure conditions, motivating a standardized, geometry-controlled evaluation^18^.

ISO 10993-5^24^ and ISO 10993-12^25^ provide internationally harmonized conditions for extraction, treatment, and cytotoxicity testing of medical devices, and recommend polyurethane containing zinc diethyldithiocarbamate (ZDEC) or zinc dibutyldithiocarbamate (ZDBC), and polyethylene as reference materials to verify assay performance^26,27^.

However, metallic molybdenum has not yet been systematically characterized under these guidelines in human osteoblast-like cells. Furthermore, ISO 10993-12 does not specify sterilization requirements for reference materials, leaving a procedural gap that may affect assay validity and reproducibility — both issues the present study directly addresses.

Osteoblasts, as the primary bone-forming cells and modulators of local immune responses, are a critical cell type to consider when evaluating a candidate implant material. Yet their molecular response to molybdenum remains poorly characterized. While the transcriptomic profile of osteoblasts on titanium under various surface conditions has been well described^28–32^, providing a valuable reference framework, equivalent data for molybdenum are absent. Prior studies on macrophages have reported upregulation of pro-inflammatory cytokines such as TNF-α and IL-1β upon molybdenum exposure, suggesting a potential inflammatory signal^33,34^; however, whether osteoblasts exhibit a similar or distinct molecular response and the resulting consequences for osseointegration have not yet been investigated.

This study therefore pursues two complementary aims. First, we evaluate the cytotoxicity of metallic molybdenum using hFOB 1.19 osteoblasts in both extract-based and direct-contact assays conducted in strict accordance with ISO 10993-5/-12, benchmarking Mo against Ti Grade 2 and WE43 Mg alloy as non-resorbable and bioresorbable clinical comparators, respectively^35,36^, and against ISO-recommended reference materials including an assessment of sterilization effects. As molybdenum is ultimately intended for translation into a medical device for animal and clinical use, the study was designed to generate high-quality, reproducible data essential for safe and regulated clinical translation. Second, we perform RNA sequencing to generate an unbiased, transcriptome-wide profile of osteoblasts cultured directly on molybdenum compared to tissue culture polystyrene controls, aiming to identify material-induced molecular processes relevant to bone healing and implant safety. Together, these data establish a standardized in vitro safety profile and molecular framework to guide the further preclinical development of molybdenum as a resorbable implant material.

## Materials and methods

### Test materials

High-purity molybdenum sheets (99.97% purity, Plansee SE, Austria) served as the primary test material. To verify assay responsiveness, sheets of polyurethane reference standards recommended by ISO 10993-5 were included: Reference Material A (RM-A, highly toxic, polyurethane containing 0.1% ZDEC), Reference Material B (RM-B, mildly toxic, polyurethane containing 0.25% ZDBC), and Reference Material C (RM-C, non-cytotoxic polyethylene control), all sourced from the Hatano Research Institute, Food and Drug Safety Center, Japan. For comparison with clinically established biomaterials, Titanium Grade 2 sheets (Goodfellow, UK) and the biodegradable magnesium alloy WE43 disks (MeoTech, Germany) were included as benchmark materials. All materials were used in their received state. All test material sheets measured 10 × 15 mm with a thickness of < 0.5 mm (total surface area ~ 3 cm^2^). The WE43 magnesium alloy disks had a diameter of 12.4 mm and a height of 1 mm (total surface area ~ 2.4 cm^2^). Additionally, sodium molybdate dihydrate (for synthesis, Merck, Germany) was used to determine LC_50_ values for molybdate ions.

### Sterilization procedures

All test materials were sterilized before biological testing to ensure aseptic handling and to prevent microbial interference with cytotoxicity results. Molybdenum sheets and the polyurethane reference materials were sterilized either using gamma irradiation, autoclaving, or ethanol treatment as indicated. Titanium Grade 2 and the magnesium alloy WE43 were sterilized exclusively by autoclaving. Following sterilization, all samples were handled aseptically throughout extract preparation and direct contact testing. For gamma irradiation, the materials were sealed in Tyvek® sterilization pouches (SteriTech, Germany) and sent to an external certified service provider (BBF Sterilisationsservice GmbH, Kernen, Germany). Irradiation was performed with a Cobalt-60 source, delivering a dose exceeding 25 kGy, ensuring effective sterilization. Autoclaving was performed at 121 °C for 20 min under saturated steam conditions. Ethanol sterilization consisted of immersing the materials in 80% ethanol, followed by sterile air-drying under laminar flow conditions.

### Cell culture

Human osteoblast-like hFOB 1.19 cells (ATCC CRL-3602) were cultured in a medium consisting of a 1:1 mixture of DMEM (Carl Roth, Germany) and Ham’s F12 (Pan-Biotech, Germany), both without phenol red, supplemented with 10% fetal calf serum (FCS) (Thermo Fisher, USA), 2.5 mM L-glutamine (Carl Roth, Germany), and 0.3 mg/mL G418 (Carl Roth, Germany) (“standard medium”). Cultures were maintained at 34 °C in a humidified atmosphere of 5% CO_2_ and routinely subcultured at 80% confluence. For passaging, cells were washed with PBS without calcium and magnesium (Carl Roth, Germany) and detached using 0.25% trypsin/EDTA (Pan-Biotech, Germany). After centrifugation at 300 × *g* for 3 min, the cell pellet was resuspended in pre-warmed medium and transferred to new culture vessels at appropriate densities for further use. Cells were manually counted using a 1:1 dilution with 0.4% trypan blue (Thermo Fisher, USA), and viability was determined as the ratio of viable to total cells. Cell line authentication and mycoplasma testing were performed at the beginning of the study (Eurofins, Germany) to confirm identity and the absence of mycoplasma contamination.

### Extract preparation

Extracts were prepared in accordance with ISO 10993-12, using a surface-area-to-volume ratio of 3 cm²/mL. Sterile material samples were placed into the wells of a 24-well plate (Sarstedt, Germany) and covered with 1 mL of extraction medium. The extraction medium was a 1:1 mixture of DMEM and Ham’s F12, supplemented with 5% FCS. Samples were incubated for 72 h at 37 °C and 5% CO_2_ under static conditions; the entire surface remained fully submerged throughout. After incubation, extracts were collected and used directly for cytotoxicity testing. They were applied either undiluted (100%) or as serial dilutions (50%, 25%, 12.5%). To further characterize the extraction conditions, pH values were measured with a pH meter (VWR pH1100L) at 20.5 °C after pooling the extracts of five specimens per test material.

### 2,3-Bis(2-methoxy-4-nitro-5-sulfophenyl) 2 H-tetrazolium-5-carboxanilide (XTT) assay

A density of 8000 hFOB 1.19 cells per well was seeded in 96-well plates (Sarstedt, Germany) in standard culture medium. After a 24 h attachment period at 34 °C and 5% CO_2_, the culture medium was removed and replaced with the respective material extracts, which had been diluted to the desired concentrations using the assay medium consisting of phenol red-free MEM (Thermo Fisher, USA) supplemented with 10% FCS, 1% L-glutamine, and 0.3 mg/mL G418. Extracts were mixed directly into this medium to achieve the final test concentrations, then added to the cells (*n*≥4 biological replicates per dilution in each experiment). Negative control wells received undiluted RM-C extract and served as the vehicle control. To determine the LC_50_ values of sodium molybdate, the cells were incubated with 0.1, 1, 3 or 10 mM sodium molybdate in culture medium (*n* = 6 biological replicates per concentration in each experiment). After 24 h of incubation with the extracts or sodium molybdate-containing medium, the XTT/PMS working solution was prepared: XTT powder (Thermo Fisher, USA) was dissolved in pre-warmed MEM (56 °C) at a concentration of 1 mg/mL. PMS (phenazine methosulfate; Thermo Fisher, USA) was dissolved in PBS without calcium and magnesium to prepare a 5 mM stock solution. Shortly before use, PMS stock was added to the XTT stock to reach a final PMS concentration of 25 µM. 50 µL of XTT/PMS working solution was added directly to each well without removing the extract-containing medium, resulting in a final reaction volume of 150 µL. Plates were incubated for 4 h at 34 °C and 5% CO_2_. After incubation, 100 µL from each well was transferred into a new 96-well plate, and absorbance was measured at 450 nm using a microplate reader (BioTek Epoch 2). Cell viability was calculated relative to negative control cells.

### Neutral red uptake (NRU) assay

Cells of the hFOB 1.19 line were seeded at 8000 cells per well in 96-well plates using standard culture medium and incubated for 24 h at 34 °C and 5% CO_2_. After attachment, the medium was removed, and the material extracts were added. Extracts were diluted with assay medium, which consisted of DMEM/ Ham’s F12 supplemented with 5% FCS, 1% L-glutamine, and 0.3 mg/mL G418. Cells were then incubated with the extract dilutions (*n*≥4 biological replicates per dilution in each experiment) or sodium molybdate-containing culture medium (*n* = 6 biological replicates per concentration in each experiment) for 24 h at 34 °C. For dye preparation, a neutral red (NR) stock solution was prepared by dissolving 0.02 g of neutral red (Sigma-Aldrich, USA) in 5 mL of dH_2_O, then aliquoted and stored in the dark. Before use, the NR medium was prepared by adding the NR stock to DMEM/ Ham’s F12, incubating overnight at 37 °C, and then centrifuging at 600 × *g* for 10 min to remove residual crystals. After the exposure to the extract, cells were gently washed with prewarmed PBS. Then, 100 µL of NR medium was added to each well and the plates were incubated for 3 h at 34 °C. Cells were washed again with PBS, and dye extraction was performed by adding 150 µL of a desorption solution composed of Ethanol: dH_2_O: acetic acid (50:49:1, v/v/v). Plates were shaken at 1000 rpm for 10 min to release the internalized dye, after which absorbance was measured at 540 nm to determine cell viability relative to the vehicle control (RM-C).

### Direct-contact assay

Cells of the hFOB 1.19 line were seeded in 6-well plates (Sarstedt, Germany) at a density of 1.8 × 10⁵ cells per well in 2 mL of assay medium (DMEM/Ham’s F12, 1:1; 10% FCS; 2.5 mM L-glutamine). Cells were cultured for 24 h at 34 °C with 5% CO_2_ to form a confluent monolayer. After incubation, 1 mL of medium was removed from each well to lower the liquid level and ensure stable positioning of the test materials. Sterile molybdenum sheets, Titanium Grade 2 sheets, and WE43 magnesium alloy samples, along with the reference materials RM-A and RM-C, were then carefully placed directly onto the cell layer (*n* = 3 biological replicates per per material in each experiment). All plates were incubated for an additional 24 h at 34 °C with 5% CO_2_. After incubation, the materials were carefully removed, and cell morphology was immediately examined using an inverted light microscope (Zeiss Axiovert, Germany). To reduce strong initial gas formation from magnesium degradation^8,11,37^, some test materials were additionally preincubated in cell culture medium for 96 h prior to cell exposure as indicated, representing a deviation from ISO 10993-5.

### Inductively coupled plasma optical emission spectrometry (ICP-OES)

To determine the concentration of molybdenum, inductively coupled plasma optical emission spectrometry was performed using an iCAP 7600 (Thermo Fisher Scientific). To ensure optical stability and enhance analytical sensitivity, the instrument optics were continuously purged with nitrogen for at least 12 h prior to measurements. The ICP was operated at a radiofrequency power of 1150 W with an argon plasma gas flow rate of 12 L/min. Measurements were performed in axial plasma viewing mode. Samples were introduced into the plasma using the built-in peristaltic pump and nebulized via a quartz cyclonic spray chamber designed for aqueous solutions. The nebulizer gas flow rate was set to 0.45 L/min. Instrument control, data acquisition, and signal evaluation were performed using Qtegra software. Molybdenum was detected at the characteristic emission wavelength of 202.03 nm.

### Evaluation criteria

For extract-based assays, results were interpreted following ISO 10993-5. A decrease in cell viability of more than 30% compared to the negative control was regarded as cytotoxic. This equates to a threshold of 70% viability in XTT and NRU assays. In the direct-contact assay, cells were observed under a light microscope after 24 h. Evaluation followed the grading system of ISO 10993-5, where Grade 0 indicates no reaction, Grade 1 indicates a slight reaction with some malformed or degenerated cells, Grade 2 indicates a mild reaction limited to the area under the specimen, and Grades 3 and 4 are considered cytotoxic.

### Statistical analysis

All graphs and data visualizations were generated using R (version 4.5.1). For comparisons between treatment groups and their respective controls, unpaired two-tailed Welch’s *t*-tests were performed. To account for multiple comparisons, *p*-values were adjusted using the Benjamini-Hochberg procedure with a false discovery rate (FDR) of Q = 0.05. Adjusted *p*-values below 0.05 were considered statistically significant. Data are presented as mean ± standard deviation. The number of replicates for each experiment is indicated in the corresponding figure legends. LC_50_ values were determined with R after fitting the data points with a 4-parameter log logistic model.

### RNA sequencing analysis

Cells of the hFOB 1.19 cell line were seeded on 1.5 cm^2^ pure molybdenum sheets in a 6-well cell culture plate with a seeding density of 167,000 cells/cm^2^ and cultured for 24 h at 34 °C and 5% CO_2_. As a control, cells were seeded onto the surface of wells of the polystyrene cell culture plate without molybdenum sheets and cultured under the same conditions. Per condition, *n* = 3 biological replicates were set up. After cultivation, the cells were detached from either molybdenum sheets or polystyrene well surface by trypsinization with 0.25% trypsin/0.53 mM EDTA and RNA was immediately isolated with the Quick RNA Minprep Kit (Zymo, USA). Following isolation, the RNA samples were quantified using the Qubit fluorometric assay. RNA sequencing and data analysis excluding gene enrichment analysis were conducted by Azenta Life Sciences (USA): Quality control was conducted by Fragment Analyzer to ensure RNA integrity. mRNA was purified and used for library preparation. RNA sequencing was performed with the Illumina NovaSeq using paired-end 2 × 150 bp protocol. The raw data quality was evaluated with FastQC. Sequence reads were trimmed to remove possible adapter sequences and nucleotides with poor quality using Trimmomatic v.0.36. The trimmed reads were mapped to the Homo sapiens GRCh38 reference genome available on ENSEMBL using the STAR aligner v.2.5.2b. Unique gene hit counts were calculated by using featureCounts from the Subread package v.1.5.2. Using DESeq2, a comparison of gene expression between the groups of samples was performed. The Wald test was used to calculate *p*-values and log2 fold changes. An adjusted *p*-value was calculated using the Benjamini-Hochberg method, to check for false positives. Genes with an adjusted *p*-value < 0.05 and absolute log2 fold change > 1 were called as significantly differentially expressed genes for each comparison. Gene ontology enrichment analysis was performed using ShinyGO v.0.85^38^.

## Results

### Effects of sterilization on the cytotoxicity of polyurethane reference materials

**Fig. 1:**
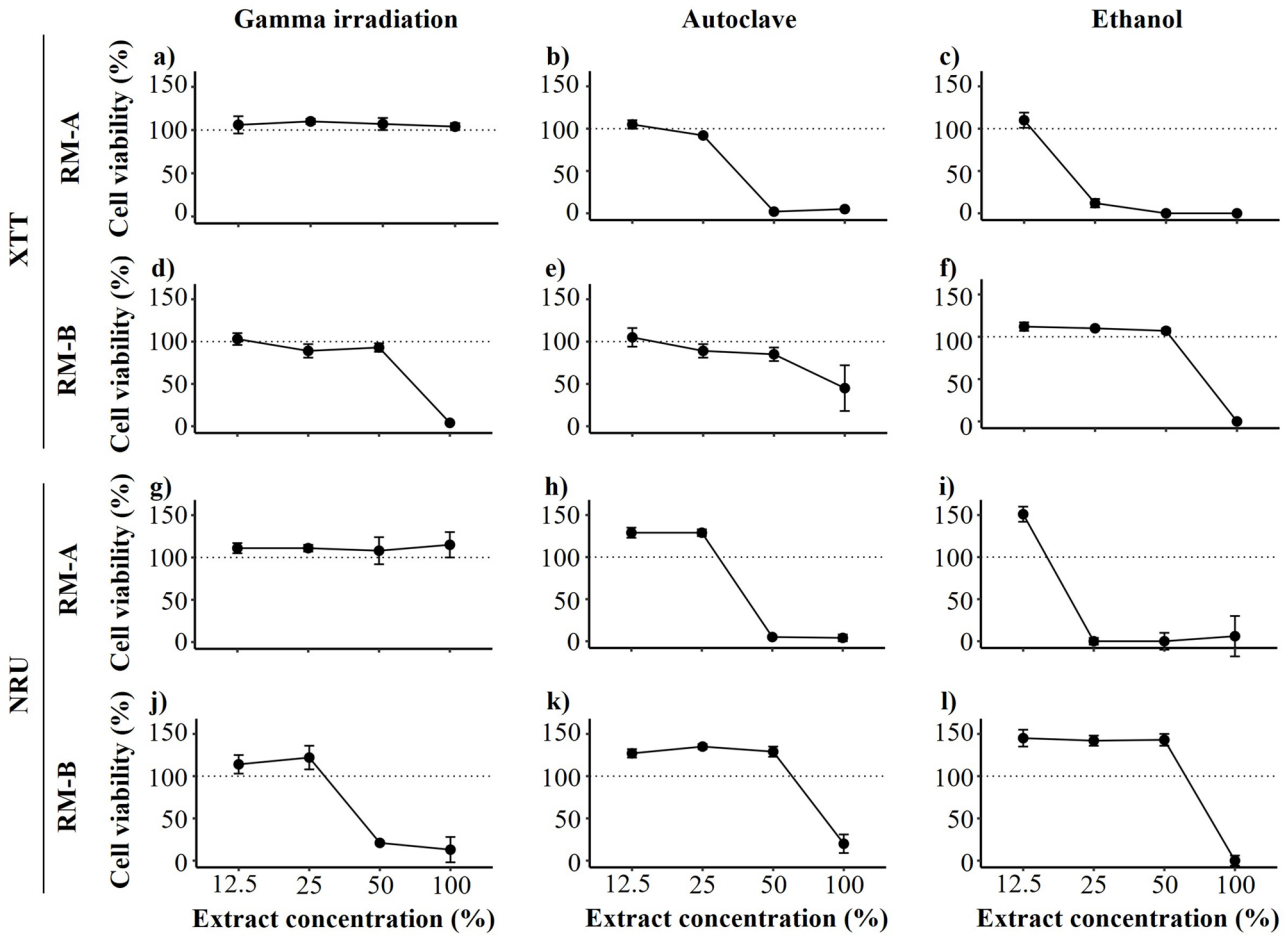
Influence of sterilization method on the cytotoxicity of reference materials RM-A and RM-B in hFOB 1.19 cells determined with the XTT (**a–f**) or NRU (**g–l**) assay respectively. Cells were exposed to extracts of RM-A (**a–c, g–i**) and RM-B (**d–f, j-l**) prepared after gamma irradiation (**a, d, g, j**), autoclaving (**b, e, h, k**), or ethanol treatment (**c, f, i, l**). Extracts were applied at concentrations of 12.5%, 25%, 50% and 100% for 24 h. Cell viability was quantified and is shown as percentage relative to the negative control (RM-C). Data represent mean ± SD from three independent experiments.

To establish a standardized sterilization protocol for the reference materials, the effects of gamma irradiation, autoclaving, and ethanol treatment on the cytotoxicity of RM-A and RM-B were assessed. The cytotoxic profile of RM-A was markedly dependent on the sterilization method (Fig. 1). Gamma-irradiated RM-A extracts yielded cell viability values close to 100% in both the XTT and NRU assays across all tested concentrations (12.5–100%), with no detectable reduction in viability, indicating complete abrogation of cytotoxic activity. In contrast, autoclaved and ethanol-treated RM-A extracts induced a concentration-dependent decline in cell viability, with more pronounced effects at higher concentrations. RM-B showed comparable cytotoxicity profiles regardless of the sterilization method applied. These results demonstrate that gamma irradiation selectively abolished the cytotoxic activity of RM-A in both assays, while the cytotoxic profile of RM-B remained largely unaffected by the sterilization procedure, underscoring the importance of sterilization method selection when using reference materials in standardized cytotoxicity testing.

### Cytocompatibility of molybdenum in extract and direct-contact assays

**Fig. 2:**
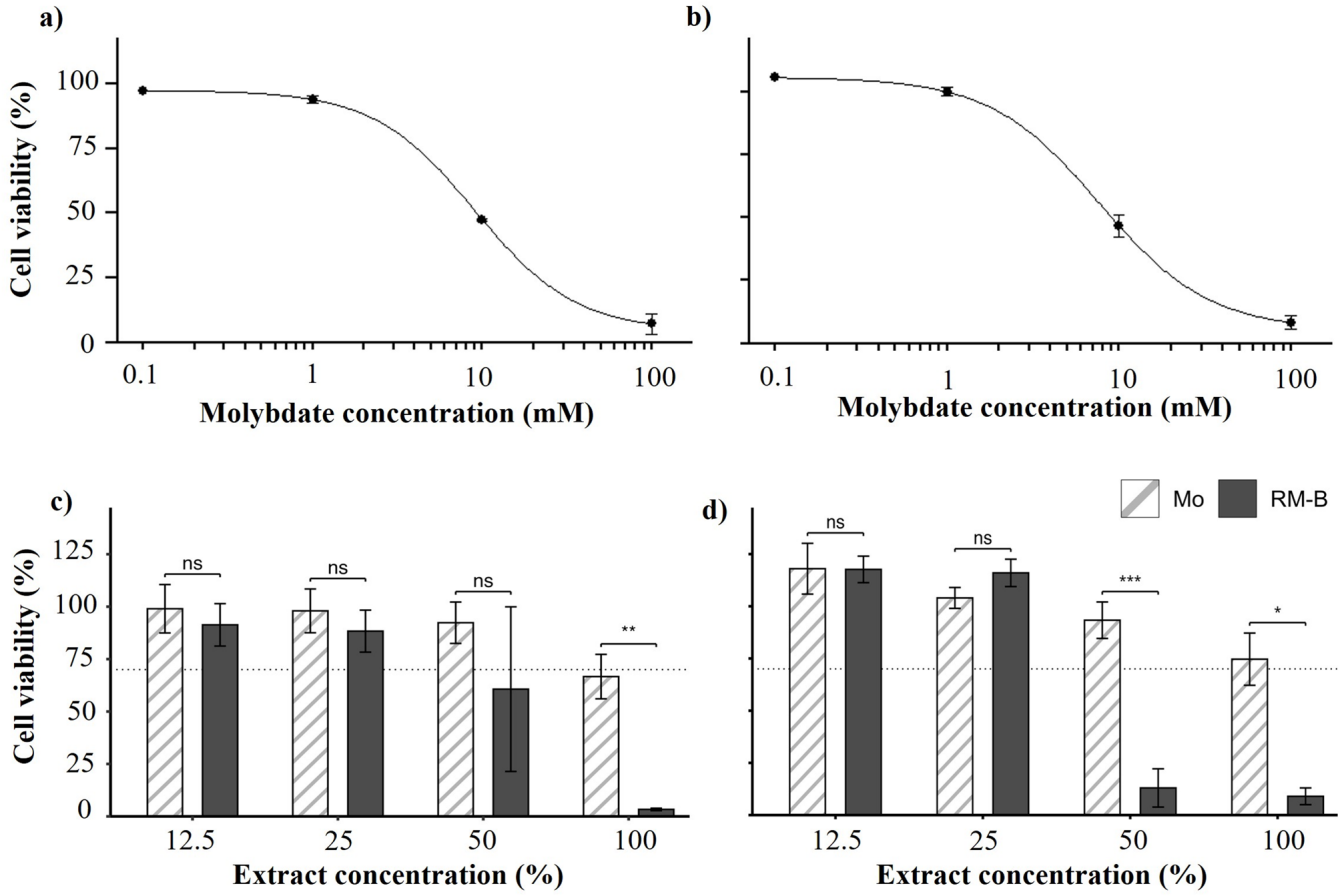
Cytotoxicity of sodium molybdate (**a, b**), molybdenum and RM-B extracts (**c, d**) in hFOB 1.19 cells. Cell viability was determined using the XTT assay (**a, c**) and NRU assay (**b, d**) after 24 h incubation with sodium molybdate, Mo and RM-B extracts at the indicated concentrations. 100% extract corresponding to a Mo concentration of 2.3 ± 0.3 mM (mean ± SD). The dotted line marks the ISO 10993-5 threshold for cytotoxicity (70%). Data represent mean ± SD from three independent experiments. Statistical analysis was performed using an unpaired two-tailed *t*-test followed by Benjamini-Hochberg correction with a false discovery rate of 0.05. Significance levels are based on adjusted *p*-values (*p < 0.05, **p < 0.01, ***p < 0.001, ns = not significant). Molybdenum and RM-B had been sterilized by gamma irradiation.

Both sodium molybdate and metallic molybdenum were evaluated for cytocompatibility with the osteoblast cell line hFOB 1.19, as the soluble salt allows direct assessment of ion-mediated toxicity, whereas with the metallic form, the corrosion process and the associated ion release were simulated under extract-based and direct-contact conditions to more closely resemble the clinical situation. Sodium molybdate induced a concentration-dependent reduction in viability within the millimolar range, yielding LC_50_ values of 9.3 mM (XTT) and 9.1 mM (NRU) (Fig. 2a, b).

The cytotoxic potential of metallic molybdenum was evaluated using both extract-based and direct-contact assays. In extract experiments, cell viability was assessed by XTT and NRU assays across four extract concentrations (12.5%, 25%, 50%, and 100%; Fig. 2c, d). Molybdenum extracts demonstrated high cytocompatibility, with viability remaining above the ISO 10993-5 threshold at concentrations up to 50% and declining moderately to values at or slightly below the threshold of 70% only with the undiluted 100% extract, corresponding to a molybdenum concentration of 2.3 ± 0.3 mM (219 ± 28 mg/L) as determined by ICP analysis.

The cytotoxic reference material RM-B induced a pronounced, concentration-dependent reduction in viability, reaching near-complete loss of metabolic activity at 100% extract. While no significant differences between molybdenum and RM-B were detected at the lower concentrations of 12.5% and 25%, molybdenum yielded significantly higher viability at 100% extract, further highlighting its favorable cytocompatibility profile. Consistent trends were observed in the NRU assay, where molybdenum maintained high viability at lower concentrations with only moderate reductions at 50% and 100% extract, while RM-B caused a strong and progressive decline in cell viability.

Direct-contact experiments yielded consistent results (Fig. 4). Following 24 h of culture, hFOB 1.19 cells grown on metallic molybdenum remained well-attached, displayed typical osteoblast-like morphology, and formed a continuous monolayer comparable to that observed on the non-cytotoxic reference material RM-C, corresponding to Grade 0 cytotoxicity per ISO 10993-5. In contrast, RM-A induced pronounced morphological alterations - including cell rounding, detachment, and monolayer disruption - indicative of severe cytotoxicity (Grade 4).

### Comparative cytotoxicity assessment of molybdenum, titanium, and magnesium

**Fig. 3:**
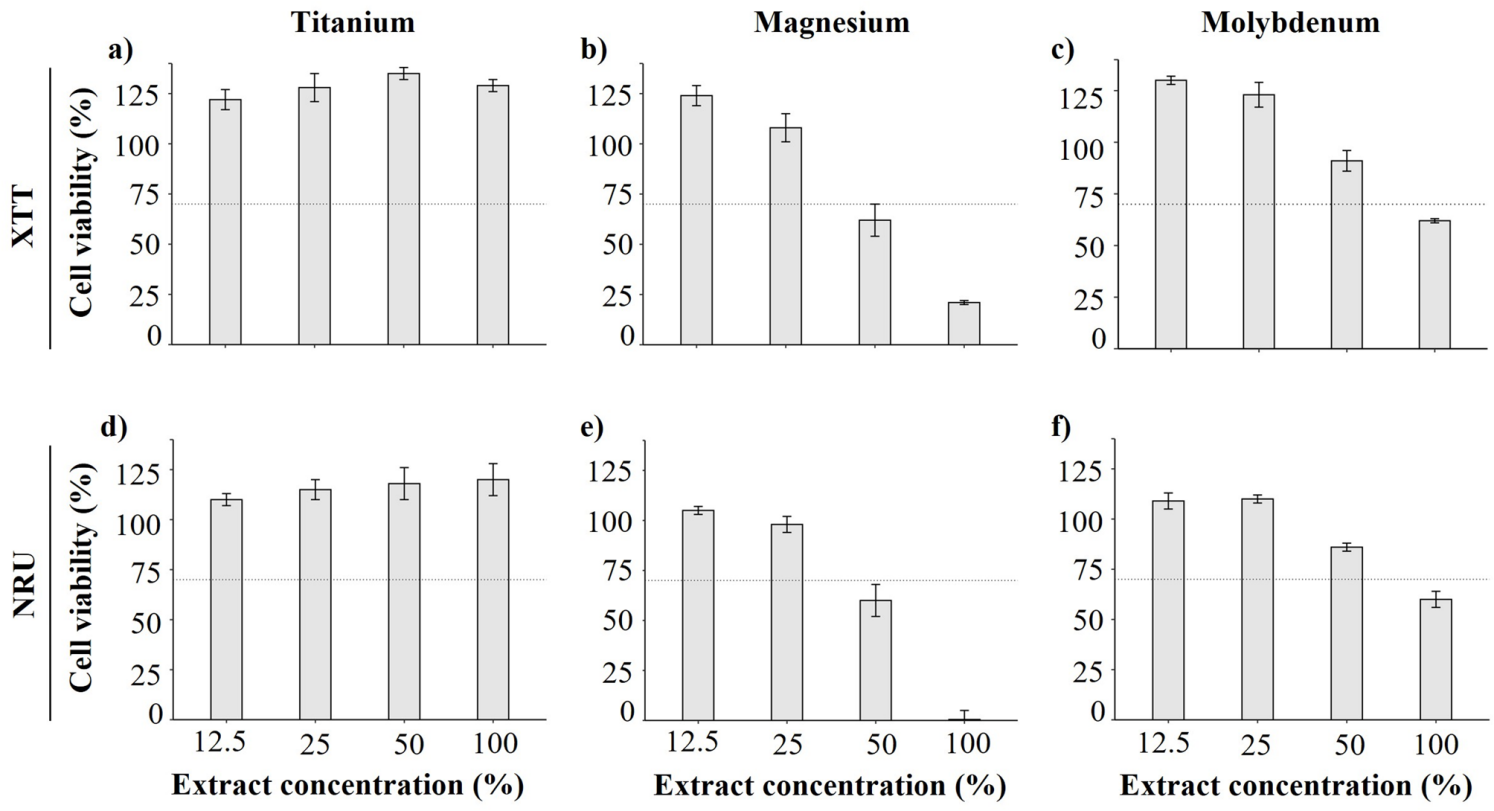
Comparison of cytotoxicity of titanium (**a, d**), magnesium (**b, e**), and molybdenum (**c, f**) extracts using XTT (**a–c**) and NRU (**d–f**) assay. Cell viability of hFOB 1.19 cells after 24 h exposure to four extract concentrations (12.5%, 25%, 50% and 100%) was analyzed. Data represent mean ± SD from three independent experiments. All materials had been sterilized by autoclaving (121 °C, 20 min).

Extract-based and direct-contact assays were conducted to compare the cytotoxic responses of molybdenum, titanium, and magnesium in hFOB 1.19 cells following 24 h of exposure. In extract-based assays (Fig. 3), titanium maintained high cell viability across all tested concentrations in both the XTT and NRU assays, with no detectable cytotoxic effects. Magnesium extracts induced a pronounced, concentration-dependent decline in viability, with values falling below the ISO 10993-5 threshold of 70% at 50% and 100% extract concentrations.

Molybdenum displayed an intermediate response: viability remained above 70% at lower extract concentrations and showed moderate reductions at 100% extract, reaching approximately 60% in both assays. Across all concentrations, molybdenum consistently yielded higher viability than magnesium, further supporting its favorable cytocompatibility profile relative to this bioresorbable comparator.

To further assess whether the observed cytotoxic effects were accompanied by changes in the extraction medium, pH values of extracts were measured. For molybdenum, titanium, RM-B and RM-C, pH values remained within a narrow range of approximately 7.5–7.9 across both extract concentrations. In contrast, magnesium extracts showed higher pH values of 8.1 and 8.4 for the 100% and 50% extracts, respectively, indicating stronger alkalinization of the extraction medium by magnesium.

**Fig. 4:**
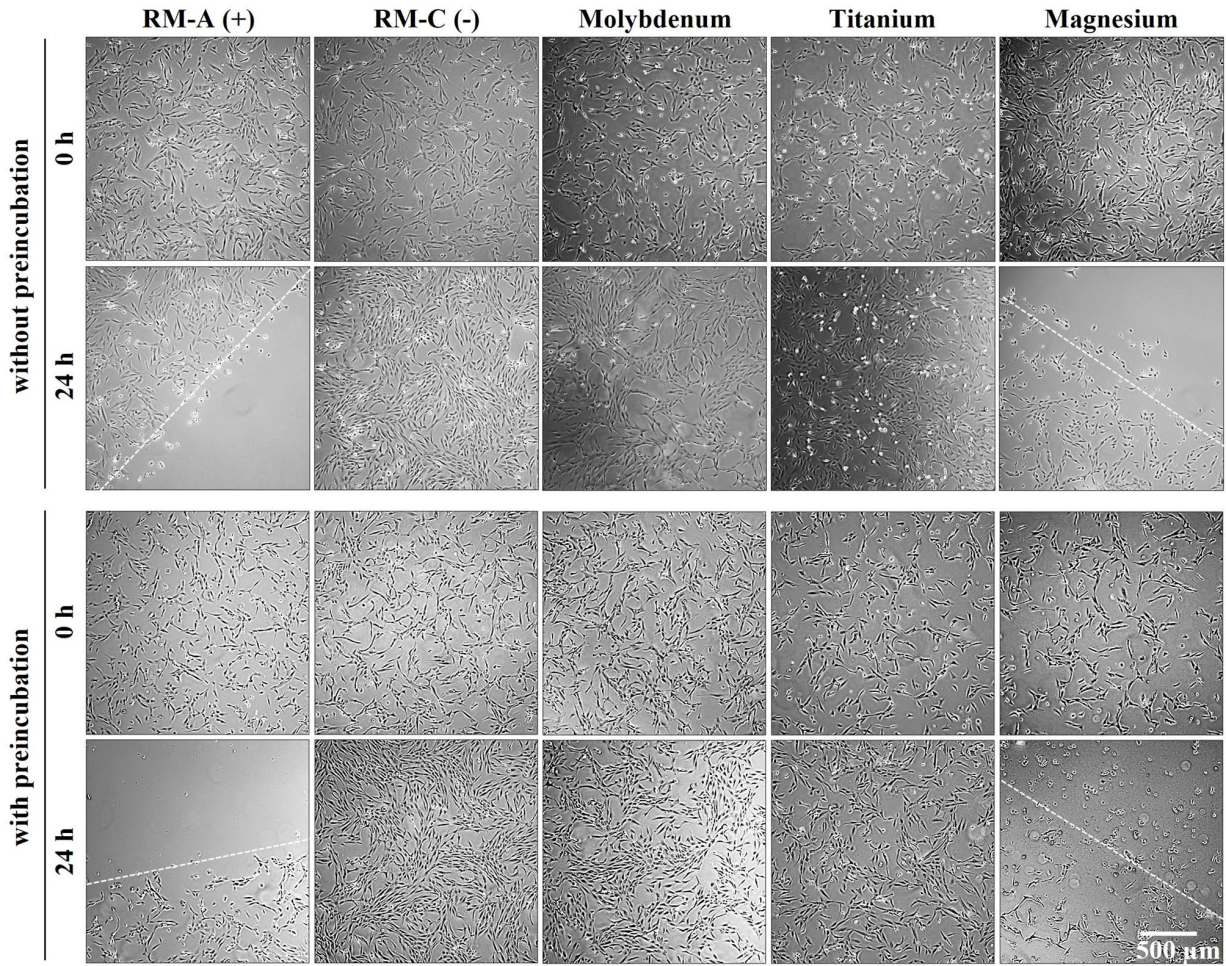
Morphological assessment of hFOB 1.19 cells after direct contact with molybdenum, reference and benchmark materials. Light microscopy images were acquired at 0 h and after 24 h of direct contact with materials, with one set of materials preincubated with cell culture medium for 96 h prior to cell contact (bottom). RM-A served as positive control and RM-C as negative control; all materials were sterilized by autoclaving (121 °C, 20 min). Images are representative of three biological replicates. Zones of growth inhibition are indicated by dotted white lines as a guide for the eye.

In direct-contact assays, both titanium and molybdenum supported intact, confluent cell monolayers after 24 h irrespective of additional preincubation, with cells maintaining normal osteoblast-like morphology and stable adhesion, corresponding to Grade 0 per ISO 10993-5 (Fig. 4). Magnesium, by contrast, resulted in reduced cell density, partial (with preincubation) or complete (without preincubation) detachment, and monolayer disruption, indicative of severe cytotoxicity (Grade 4). These qualitative observations were fully consistent with the extract-based findings, reinforcing the comparative cytotoxicity profile across all three materials. Direct-contact assays with preincubated materials yielded comparable results for all materials except magnesium, with cell morphology and growth inhibition patterns consistent with those observed without preincubation, suggesting that preincubation did not substantially affect the observed cytotoxic response under the tested conditions.

### Transcriptomic profile of hFOB 1.19 in response to molybdenum

To further characterize the interaction between metallic molybdenum and osteoblasts and to investigate the molecular processes underlying the mild cytotoxic response observed at high molybdenum extract concentrations, we performed RNA-seq analysis on hFOB 1.19 cells cultured directly on molybdenum plates for 24 h, comparing their gene expression profile to cells grown on standard tissue culture polystyrene.

**Fig. 5:**
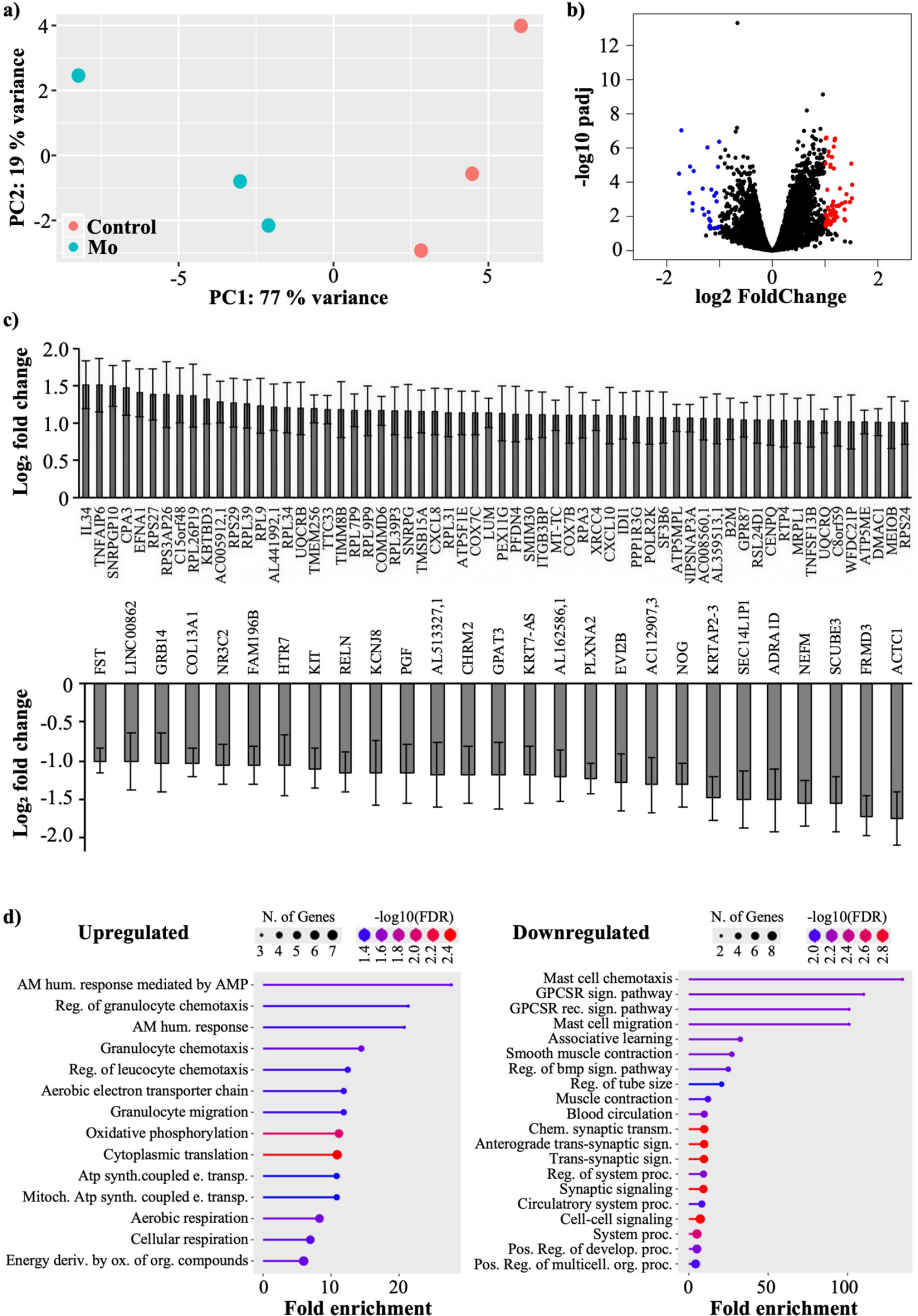
Characterization of transcriptomic response to molybdenum. (**a**) Principal Component Analysis. Red: cells cultured on plastic cell culture plate, turquoise: cells cultured on molybdenum (*n* = 3 biological replicates per material). (**b**) Volcano Plot (red: significantly overexpressed genes, blue: significantly underexpressed genes). Figure (**a, b**) were generated by Azenta Life Sciences. (**c**) Differential gene expression of significantly overexpressed (top) and significantly underexpressed (bottom) genes. Error bars indicate standard errors (**d**) Gene ontology enrichment analysis on upregulated DEGs and downregulated DEGs of biological processes, Figures generated with ShinyGO v.0.85 (https://bioinformatics.sdstate.edu/go/). Molybdenum sheets had been sterilized by autoclaving (121 °C, 20 min). (PC = principal component; padj = p-adjusted value; AM = antimicrobial; hum = humoral; AMP = antimicrobial peptides; Reg. = regulation; synth. = synthesis; e. = electron; transp. = transport; mitoch. = mitochondrial; deriv. = derivation; ox. = oxidation; org. = organic; GPCSR = G-protein coupled serotonin receptor; sign. = signaling; cyc. = cyclic; nucl. = nucleotide; mes. = messenger; chem. = chemical; transm. = transmission; proc. = process; Pos. = positive; FDR = false discovery rate).

We obtained an average of 24.6 ± 0.7 and 22.7 ± 0.7 million reads (mean ± standard deviation; *n* = 3) from cells cultured on magnesium and on tissue culture plastic, respectively. Of these, 96.55 ± 0.08% and 98.83 ± 0.09% (mean ± standard deviation; *n* = 3) mapped uniquely to the human reference genome. Principal component analysis confirmed consistent clustering of biological replicates within each group (Fig. 5a). Of 14,928 genes detected, 89 were significantly differentially expressed under stringent criteria (adjusted *p <* 0.05, |log₂ fold change| > 1), comprising 62 upregulated and 27 downregulated genes (Fig. 5b, c). Gene ontology enrichment analysis of the upregulated gene set identified 14 enriched biological process terms, broadly grouping into three functional categories: immune response, mitochondrial activity, and protein biosynthesis, while analysis of the downregulated gene set returned 20 enriched biological process terms, predominantly associated with osteoblast signaling, proliferation, and differentiation (Fig. 5d).

To further examine effects on bone-forming function, gene ontology enrichment analysis was performed for the term “ossification,” returning 43 differentially expressed genes (adjusted *p <* 0.05), of which 26 were downregulated and 17 upregulated. Importantly, canonical osteogenic markers — including RUNX2, COL1A1, COL3A1, ALPL, SPP1 (osteopontin), BGLAP (osteocalcin), and TNFSF11 (RANKL) — were not differentially expressed, suggesting that core osteoblast identity and matrix-forming capacity are preserved under the tested conditions.

## Discussion

The present study evaluated the cytotoxicity of molybdenum under standardized conditions per ISO 10993-5^24^ and ISO 10993-12^25^ using extract-based assays and a direct contact test in human osteoblast-like hFOB 1.19 cells and complemented this evaluation with a transcriptomics analysis.

In extract-based assays, molybdenum consistently supported high cell viability across dilutions (12.5, 25, and 50% extract). At 100% extract concentration, viability decreased to approximately 60%, falling below the 70% cytotoxicity threshold and thus indicating mild cytotoxicity without complete loss of metabolic activity or membrane integrity. We hypothesize that the observed reduction in cell viability may be attributable to oxidative stress previously described upon molybdenum exposure in hepatocytes and epithelial cells^39,40^ and/or due to genotoxicity observed in plant cells^41^. In the direct-contact assay, cells adjacent to molybdenum maintained normal morphology and an intact monolayer after 24 h, consistent with a non-cytotoxic response. These findings align with previous reports classifying molybdenum among the least cytotoxic implant-relevant metals, with LC_50_ values exceeding 7 mM in other human osteoblast models and no significant pro-inflammatory cytokine release at subtoxic concentrations^17,22^. In the present study, sodium molybdate yielded comparable LC_50_ values in hFOB 1.19 cells, indicating a similar susceptibility to molybdate ions as previously reported for other osteoblast cell lines. The mild viability reduction at 100% extract concentration likely reflects accumulation of molybdate to elevated concentrations under static extraction conditions, as the 72 h incubation period promotes higher local ion concentrations than those expected in dynamic in vivo environments with continuous fluid clearance, where favorable tissue compatibility has been reported^17,23^. ICP-OES confirmed molybdate concentrations of approximately 2.3 mM in the 72 h extracts, coinciding with the observed viability reduction and suggesting this concentration range as a relevant cytocompatibility boundary under standardized conditions. Additional factors beyond molybdate ions may contribute to the cytotoxicity observed in molybdenum extracts, since a molybdate concentration of 2.4 mM alone is expected to reduce cell viability only to approximately 80–90% (Fig. 2a, b).

Comparative assessment highlighted clear material-specific behaviors. Titanium maintained high viability and preserved cell morphology in both assay formats, consistent with its established biocompatibility^42,43^. Magnesium exhibited pronounced concentration-dependent cytotoxicity, attributable to rapid degradation-associated increases in pH and ionic strength^8,44^. Molybdenum displayed an intermediate biological profile, with cellular responses in direct contact closer to titanium than to magnesium. The extreme cytotoxic effects of magnesium also illustrate a well-recognized limitation of standardized in vitro testing for rapidly degrading metals, where extracts may reach unphysiological ion concentrations unlikely to occur in vivo^8,44^. All results should therefore be interpreted within the defined framework of standardized testing and approaches to achieve more physiologically relevant conditions are sought after^11,45^.

A relevant methodological observation concerns the effect of gamma irradiation on standardized polyurethane reference materials containing zinc dithiocarbamates. Sterilization of the strongly cytotoxic reference (RM-A) resulted in loss of expected biological activity, demonstrating that irradiation can alter material properties and confound cytotoxicity assessments. This underlines the importance of validating reference material performance under the specific sterilization conditions applied in a given study.

In cells cultured on molybdenum substrates, transcriptome analysis revealed strong enrichment of gene ontology terms associated with immune cell recruitment and chemokine-mediated signaling - most prominently “Antimicrobial humoral response mediated by antimicrobial peptides” and “Regulation of granulocyte chemotaxis”. Key upregulated genes within these sets included TNFAIP6, IL34, CXCL8, and CXCL10, encoding cytokines that regulate diverse immune cell populations. Notably, IL34 and TNFAIP6 are both established regulators of bone homeostasis with context-dependent roles in osteoclast activation, differentiation, and osteoblastogenesis^46–49^, while CXCL8 is a chemokine whose upregulation has been shown to enhance osteoblast-mediated osteoclastogenesis via IL-6 signaling^50^. The upregulation of genes involved in immune response is in accordance with macrophage studies reporting an increased expression of pro-inflammatory mediators upon contact with molybdenum^34^. Notably, markers of acute inflammation such as IL-1β and IL-6 were not significantly upregulated, indicating a controlled signaling response rather than an acute inflammatory reaction. In parallel, the upregulation of mitochondrial genes encoding electron transport chain components, including cytochrome c oxidase, together with enrichment of cytoplasmic translation terms, points to an increased capacity for aerobic energy production and protein biosynthesis.

Among the downregulated genes, the most strongly enriched gene ontology terms comprised “G-protein coupled serotonin receptor signaling pathway”, “mast cell migration”, and “mast cell chemotaxis”. Within the “G-protein coupled serotonin receptor signaling pathway” term, HTR7 and CHRM2 - encoding serotonin and muscarinic acetylcholine receptors, respectively - were significantly downregulated. CHRM2, a component of the cholinergic signaling system, has been identified as a regulator of bone remodeling and osteoblast proliferation^51,52^. Among the genes driving enrichment of “mast cell migration” and “mast cell chemotaxis”, KIT and PGF were significantly downregulated; KIT encodes a receptor tyrosine kinase for stem cell factor involved in the regulation of proliferation, differentiation, and migration across multiple cell types^53,54^, while PGF encodes a growth factor known to be regulated by the osteogenic morphogen BMP-2^55^. Collectively, the downregulation of these signaling-related genes suggests reduced osteoblast proliferative capacity upon contact with molybdenum, while - as noted above - core osteogenic markers remained unaffected, indicating that fundamental bone-forming function is preserved.

The transcriptional response of osteoblasts cultured on titanium, a benchmark implant material, has been extensively characterized^28,29,32,56,57^. Previous studies report sustained expression of key osteogenic differentiation markers, such as osteocalcin, osterix, osteopontin, and collagen I and retained mineralization capacity^29,32^. Similarly, in the present study, molybdenum did not significantly change the expression of osteogenic marker genes, indicating that the extracellular matrix biosynthesis capacity is preserved upon contact with this material. To maintain consistency with the direct contact viability assays, we applied a 24 h incubation period, which may be insufficient to detect changes in osteogenic differentiation markers such as RUNX2 and ALPL. Follow-up studies with longer incubation times, combined with additional biochemical assays, are warranted to investigate long-term cellular responses to molybdenum exposure. Osteoblasts cultured with magnesium, used as a bioresorbable implant material, are reported to exhibit an enhanced expression of the osteogenic markers ALP and osteocalcin in vitro, indicating active bone formation^58,59^.

In conclusion, molybdenum demonstrates high cytocompatibility under standardized ISO 10993-5/-12 conditions, with cytotoxic effects confined to molybdate concentrations in the millimolar range. Transcriptome analysis of osteoblasts in direct contact with molybdenum suggests increased immunostimulatory signaling as well as enhanced aerobic energy metabolism and protein biosynthesis, while expression of osteogenic differentiation markers was not impaired. Taken together, these findings provide a robust preclinical basis supporting the further evaluation of molybdenum as a bioresorbable metallic material for implant applications in the bone compartment, while emphasizing that local molybdate ion concentrations at the implant-tissue interface must remain below cytotoxic thresholds to ensure biocompatibility.

## Data Availability

All data supporting the findings of this study are openly available in the Zenodo repository (10.5281/zenodo.19497956).
